# Global Irradiation in Children Treated for Hydrocephalus and Its Change over Time—A Single Institutional Analysis

**DOI:** 10.3390/children9071062

**Published:** 2022-07-16

**Authors:** Lukas Schabl, Julia Küppers, Tobias Jhala, Hermann Winicker, Peter Esslinger, Markus Lehner

**Affiliations:** Department for Pediatric Surgery, Lucerne Cantonal Hospital, 6000 Lucerne, Switzerland; juleskueppers@gmail.com (J.K.); tobias.jhala@luks.ch (T.J.); hermann.winicker@salk.ch (H.W.); peter.esslinger@luks.ch (P.E.); markus.lehner@luks.ch (M.L.)

**Keywords:** shunt, hydrocephalus, radiation, ventriculoperitoneal, MRI, CT

## Abstract

Radiation exposure early in life is associated with greater incidences of malignancy. Our goal was to quantify radiation exposure in shunt-treated hydrocephalus patients and study changes in the diagnostic modalities used. A single-center, retrospective analysis was performed, and 41 children treated for hydrocephalus using an adjustable ventriculoperitoneal shunt were identified. Diagnostics associated with hydrocephalus and other comorbidities were analyzed and radiation exposure was calculated. During 330.09 total shunt years, patients were exposed to a mean hydrocephalus-associated radiation dose of 3.93 mSv (range: 0–24.38 mSv), which amounted to a mean rate of 0.49 mSv per shunt year, respectively. Radiation exposure was greatest after shunt insertion in the first year of life. A continuous change from CT scans to MRIs could be seen over the study period, such that patients who underwent shunt insertion after 2017 were not exposed to additional hydrocephalus-associated radiation during their first year of life. Nevertheless, our patients, and a few individuals especially, seemed to be at higher risk for radiation sequelae. Our results suggest that CT scans should be substituted with MRIs, which decrease overall radiation exposure and can lead to zero additional radiation exposure during the first year of life after shunt insertion.

## 1. Introduction

Multiple studies describe an association between exposure to radiation and development of diseases, with malignancy leading above others [1,2,3,4]. While some studies have looked at historical tragedies where people have been exposed to immense doses of radiation, more recent work has tried to directly quantify the risk of deleterious effects from radiation [2,3,4].

While it is commonly accepted that reducing the level of radiation exposure is reasonably achievable, radiation is still considerable in patients treated for hydrocephalus [5,6]. This partly relates to the relatively high incidences of shunt malfunction and surgical intervention and their effects on the longevity of the disease. CT scans are frequently used for shunt-treated hydrocephalus. The level of radiation caused by CT scans has so far been described in three studies, of which only one included other imaging modalities [5,6,7]. Hydrocephalus can be accompanied by comorbidities that result in further radiation from imaging. Nevertheless, the additional radiation burden from comorbidities is underrepresented in the literature.

In our study, we examined the quantifications for all the imaging modalities associated with hydrocephalus and its comorbidities in order to calculate the radiation burden using estimated radiation doses and calculations derived from a literature review.

Various ways of reducing radiation exposure have been described, among which rapid-sequencing MRI seems to be the most reasonable [8,9]. We evaluated the change from using CT scans to MRI for patients treated for hydrocephalus at a specific hospital during the study period.

Patients who were younger than 1 year old at the time of shunt insertion seem to acquire a higher radiation burden [5,6]. Therefore, we also looked at this subgroup.

## 2. Materials and Methods

**Figure 1:**
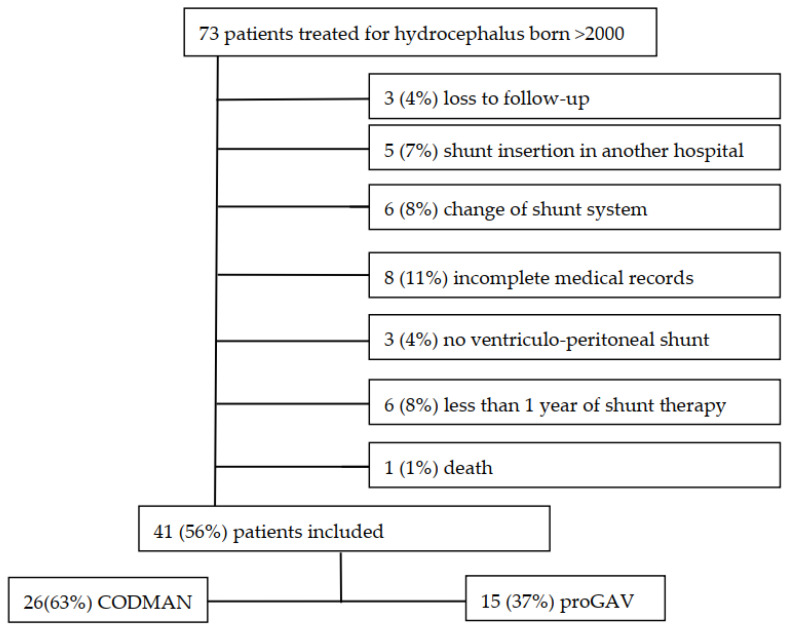
Patient recruitment.

We performed a single-center, retrospective study including pediatric patients (0–16 years) treated for hydrocephalus using a ventriculoperitoneal shunt between 2000 and 2021. All patients were treated at a level-one pediatric surgery center that was the main health care provider and the only pediatric neurosurgery unit in an area inhabited by a population of over 700,000. Patient details were identified using available databases and cross-referenced with their medical records and hospital radiology databases (PACS). Radiology databases are connected to local emergency departments and, for all the children treated, our pediatric neurosurgery unit was the first emergency contact, so it is very unlikely that imaging was performed without being registered in the database. The data recorded included: demographics, diagnosis, shunt type and insertion date, type and time of surgeries and type and time of CT, MRI and X-ray imaging. Furthermore, we differentiated the data according to whether imaging was requested to evaluate hydrocephalus or for another indication. Patients were excluded in the following cases: death, shunt insertion in another hospital, changes in the shunt system, use of shunts other than ventriculoperitoneal ones, loss of follow-up and in situ shunt times of less than 1 year (Figure 1).

For each patient, the total number of imaging procedures and the number of hydrocephalus-associated MRIs, CT scans and X-rays, along with the total time that the shunt device was used in situ, were calculated. The MRI and CT imaging procedures used to evaluate hydrocephalus were exclusively head scans. The average number of imaging sessions per shunt year was then determined. Exact radiation and effective tissue dose data were not available; therefore, we depended on previous studies and performed calculations based on an average dose of 2 mSv per cCT (CT head scan), 0.01 mSv per lateral skull X-ray and 0.1 mSv per shunt series [10,11]. CODMAN shunt systems (programmable Medis Siphon-Guard 82-3832) were implanted until 2017 and proGAV shunt systems (proGAV 1.0) after 2017. The change from CODMAN to proGAV in 2017 was a result of a change in the leadership positions in the pediatric neurosurgical unit of our institution. Direct comparison of the two shunt systems was undertaken for patients under 1 year of age. Finally, we searched for changes in the use of CT scans and MRI over time.

Both shunt systems function using a differential pressure valve, based on a “ball on spring” principle, that opens and permits the outflow of the cerebrospinal fluid (CSF) when the CSF pressure exceeds the combined resistance of the intraperitoneal pressure and the valve setting. Additionally, the proGAV shunt system makes use of a gravitational unit, which automatically increases and decreases the resistance to the CSF outflow in accordance with the patient’s vertical or supine position. Both systems can be adjusted with a magnet over the skin. However, the proGAV shunt settings can be verified using the so-called proGAV Compass, while CODMAN shunt systems are verified using skull X-rays, which display the settings.

Ethical approval for this study was obtained from the Swiss ethics committee BASEC under the ID number 2021-00640.

## 3. Results

Patients were identified during our investigation period from 2000 to 2021. A total of 73 patients were treated for hydrocephalus with a shunt. Of those, we excluded five (7%) due to shunt insertion in another hospital, three (4%) due to loss of follow-up, six (8%) due to changes in the shunt system, eight (11%) due to incomplete medical records, three (4%) due to implantation of a shunt other than a ventriculoperitoneal one and six (8%) due the shunt therapy lasting less than 1 year. One (<1%) patient died during the follow-up period from septic shock due to bacterial meningitis. Of the remaining 41 (56%) patients, 26 (63%) were treated with a CODMAN shunt system and 15 (37%) with a proGAV shunt system. Patient recruitment is summarized in Figure 1. In total, 22 (54%) patients were male, 28 (69%) were under the age of one year and 10 (24%) were aged between one and six years at the time of shunt insertion. All shunts were ventriculoperitoneal, and the occurrences of hydrocephalus were categorized as posthemorrhagic (34%), spina bifida (17%), aqueduct stenosis (17%), tumor (17%), congenital brain malformations (7%), postinfectious (2%), arachnoidal cyst (2%) and neurofibromatosis (2%).

Total shunt time in situ was 330.09 years (295.8 years for CODMAN, 34.3 years for proGAV), indicating a mean of 8.07 shunt years per patient (range: 1.06–15.8 years). The mean age at insertion was 669 days (range: 1–5366 days).

During our study period, 109 CT scans were performed, of which 78 (71%) scans were indicated to be due to hydrocephalus and were head scans (cCTs). The other 31 scans were conducted for other medical reasons and included CT scans of the abdomen (5 mSv), pelvis (5 mSv), feet (1.5 mSv), chest (8 mSv), lower extremities (3.4 mSv), petrosal bone (1.2 mSv) and spine (6 mSv), as well as PET-CT scans (21.6 mSv) [11,12,13,14,15].

**Figure 2:**
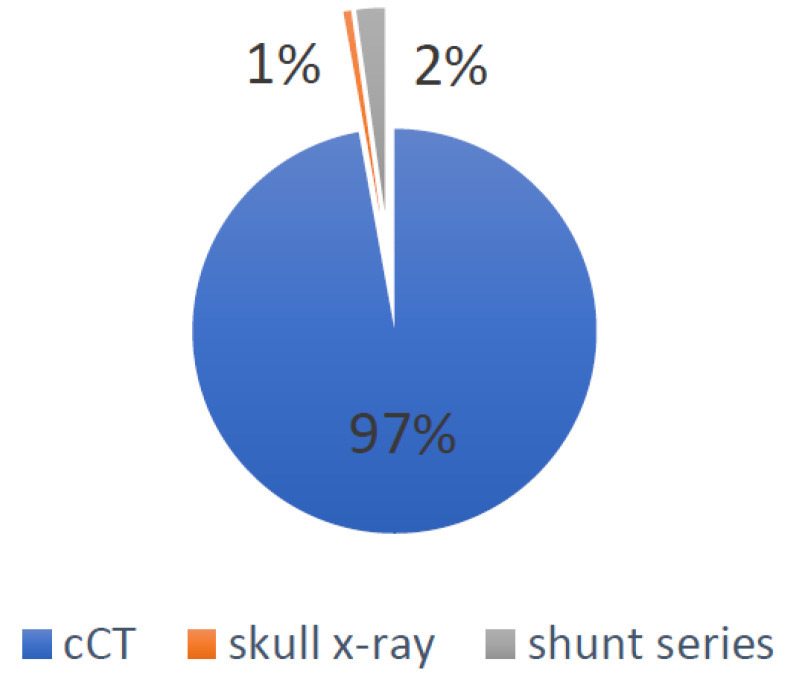
Visualization of the percentages of the different causes accounting for the global irradiation.

**Figure 3:**
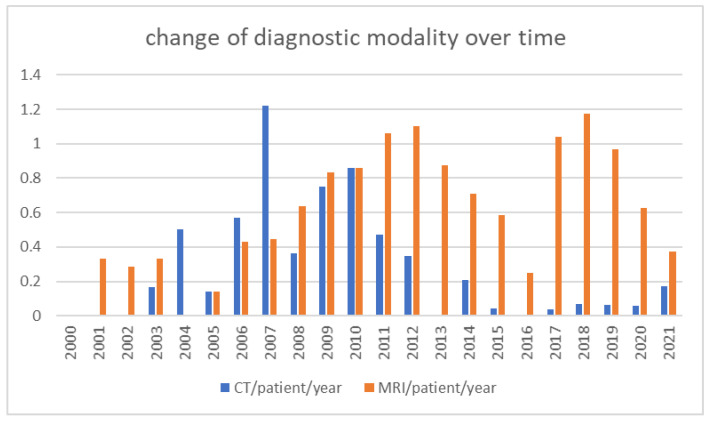
Change in diagnostic modality over time. Values present the rates of cCTs and MRIs per patient for the indicated year.

- Overall, 41 patients were exposed to a total of 78 cCTs, of which 18 (23%) were performed during the first year of life, 38 (48%) between the ages of one and six and the remaining 14 (18%) after the age of six. Patients had a mean cCT rate of 1.9 per patient (0–12) and a mean cCT rate of 0.23 per shunt year. There was a mean shunt series rate of 0.92 (range: 0–4) and, for the lateral skull X-rays used to verify shunt settings, the mean rate was 2.37 (range: 0–13). Adding together the cCTs, shunt series X-rays and lateral skull X-rays, patients were exposed to a mean radiation exposure due to hydrocephalus of 3.93 mSv (range: 0–24.38), accounting for 0.49 mSv per shunt year. During the same study period, 272 MRIs were performed on these 41 patients due to hydrocephalus, leading to a mean MRI rate of 6.63 (range: 0–17). Table 1 summarizes these results.
- For patients operated on before 2017—and therefore with a CODMAN shunt system—radiation exposure due to hydrocephalus during the first year of life was calculated as having a mean of 1.68 mSv (range: 0–18.67), taking into consideration 18 cCTs, 8.7 shunt series X-rays and 3 lateral skull X-rays. Patients operated on after 2017—and therefore with a proGAV system—were not exposed to a single cCT, shunt series X-ray or lateral skull X-ray during the first year of life, meaning no additional radiation exposure due to shunt therapy.
- Radiation exposure, considering CT scans for all locations and for indications other than hydrocephalus during the same study period (seven pelvis CT scans (5 mSv), three abdomen CT scans (5 mSv), three PET-CT scans (21.6 mSv), two foot CT scans (1.5 mSv), four chest CT scans (8 mSv), one lower limb CT scan (3.5 mSv), four spine CT scans (6 mSv) and one petrosal bone CT scan (1.2 mSv)), added up to 178.5 mSv, which was 1.85 mSv per shunt year or 4.3 msV per patient (range: 0–80.8 mSv) [11,13,14,15].
- Global irradiation was 162.64 mSv, of which 158 mSv were caused by cCTs, 3.67 mSv by shunt series X-rays and 0.97 mSv by lateral skull X-rays. The causes for the global irradiation are visualized in Figure 2.
- For all patients, the dates of the MRIs and head CTs were listed in order to evaluate the possible change during the observation period. Figure 3 visualizes this change. Between 2000 and 2010, 4.57 CTs and 4.28 MRIs per patient were performed, in comparison to 1.84 CTs and 8.75 MRIs per patient between 2011 and 2021.

## 4. Discussion

Our results show that, on average, shunt patients were exposed to 1.9 cCT scans, which amounts to 0.23 cCT scans per shunt year. Dobson et al. found that 0.65 CT scans were performed per shunt year in a cohort of 152 children with 778 shunt years [5]. The study by White et al. included 62 patients (989 shunt years) with shunt placement during the first year of life, with an average number of 16 cCT scans per patient over an average follow-up period of 16 years. There were 42 patients (633 shunt years) aged one to six years with shunt placements, with an average number of 14 scans per patient over an average follow-up period of 14 years [6]. Cohen et al. investigated the use of CT scans in an emergency department and found that, for 232 patients over a period of 10 months, the average number of cCTs performed was 2.6 [16]. Antonucci et al. included 130 patients over a 10 year observation period with a median age of 1.1 years at the beginning. Their cohort was exposed to 0.9 CTs per shunt year [7].

In comparison with the studies mentioned above, our results demonstrate a low number of cCT scans per shunt year, as displayed by Table 2. One explanation might be that we included patients with long shunt in situ times of up to 15 years. Since shunt malfunction often occurs in the first year after insertion, those patients might have lowered the overall number of cCTs per shunt year [17]. This might also explain the difference in CT rates between Cohen et al.’s study and ours, and it was already recognized as a possible factor by Antonucci et al. [7]. Nevertheless, we think that one reason for our results was the rigorous implementation of MRIs whenever feasible by also using rapid sequences/HASTE, even in the emergency setting. This can be seen in Table 2, which shows a very high number of head MRIs per shunt year compared to Antonucci et al., for example.

We found that, in patients operated on before 2017 and with a CODMAN shunt system, radiation exposure was greatest during the first year of life. For the total of 295.8 shunt years, a value of 140.92 mSv was calculated. Twenty-two patients received their shunts during the first year of life, for whom a value of 36.89 mSv was calculated. The radiation exposure during the first year of life accounted for about a fourth of the total exposure. The mean radiation dose applied to these patients during their first year of life was 1.68 mSv (range: 0–18.67), which can be compared to 0.44 mSv for all shunt years. These findings agree with the results of previous studies, which also showed increased radiation exposure in younger age groups [5,6,7]. One explanation is that clinical evaluation is more difficult in infants and younger children; therefore, imaging is used more often. Additionally, shunt placement during the first year of life is recognized as an individual risk factor for shunt malfunction [17]. Interestingly, patients in our cohort who received their shunts after 2017 as part of the proGAV system were not exposed to a single cCT scan or X-ray to evaluate their hydrocephalus or shunt during their first year of life. The change in shunt system might partially be responsible for the decrease in the use of CT scans. Furthermore, a rigorous plan involving the use of MRI scans in follow-up and preoperative assessment primarily and, whenever feasible, rapid-sequence MRI for emergency assessment was introduced.

Our results demonstrate that not all patients treated for hydrocephalus are at higher risk due to radiation exposure, but certain individuals may be. While some were not exposed to any additional radiation, the maximum radiation burden for one patient was 24.38 mSv. These findings correlate with the data presented by Dobson et al., who even described a difference as high as 42 mSv between the highest and lowest individual radiation burdens [5]. As not every hydrocephalus patient is at higher risk of radiation sequelae and following all patients does not seem realistic due to time and financial reasons, we think that it makes sense to focus on individuals in order to be aware of their additional risks in the future.

Some shunt systems still need their settings verified using lateral skull X-rays. Whilst the radiation dose (0.01 mSv) may be negligible in comparison to a cCT scan (2 mSv), we think that, by using newer shunt systems such as the proGAV 1.0 system, unnecessary radiation can be avoided [11]. There were no lateral skull X-rays performed to assess shunt settings in patients using the proGAV system, while 97 skull X-rays were performed in patients using the CODMAN system.

Children with hydrocephalus might present comorbidities, which can increase the overall radiation burden. The radiation burden due to imaging studies associated with hydrocephalus was 162.64 mSv. An additional 178.5 mSv were caused by imaging studies for comorbidities. It is worth mentioning that certain individuals were again at higher risk; e.g., one patient was exposed to three PET-CT and two chest CT scans, adding up to 80.3 mSv.

Nevertheless, these numbers demonstrate that, while most of the radiation burden is due to investigation of hydrocephalus, attempts to limit radiation exposure must not stop at cCTs but should involve the reevaluation of every imaging procedure using radiation.

While none of our patients developed a malignancy that could be traced back to radiation exposure, we do not think that we included enough patients or a long enough follow-up period to conveniently address this issue. The results from White et al.’s study also did not reveal any malignancy during the average follow-up period of 16 years in their cohort of 62 shunt-treated children [6]. Nevertheless, multiple studies with higher numbers of patients included have described a link between radiation exposure and the occurrence of malignancies mainly but also other sequelae. Mathews et al. included over 680,000 people and found an increase in the overall cancer incidence of 24% after a single CT scan and an additional 16% increase for every following scan, highlighting that the incidence rate ratio was even higher in younger patients [3]. For 60,674 diagnosed cancers, the excess cancer rate was estimated at 1%. Pearce et al. estimated that the risk of developing brain cancer tripled after exposure to 60 mGy and noted that 10,000 CT scans were responsible for one excess case of leukemia and one excess case of brain tumor development [4]. According to another study published in 2013, the 4 million CT scans per year in the US are projected to cause 4870 future cancers, and even reducing just the highest 25% of radiation doses could decrease the rate by 43% [18]. Larger prospective trials over a lifetime would be needed to quantify the increase in risk for radiation sequelae.

Our results demonstrate that the rising awareness regarding radiation sequelae has led to a change in the use of diagnostic modalities. While the use of CT scans and MRIs was about equal during the first seven years of our observation period, a move towards MRIs was noted during the following years (Figure 3). Rapid-sequence MRI has already been described as an alternative to cCTs in previous studies. Patel et al. demonstrated in a cohort of 200 children (mean age: 5.7 years) that rapid-sequence MRI produced images with excellent quality compared to cCTs. Moreover, none of the children needed sedation during the process [9]. Koral et al. emphasized the role of rapid-sequence MRI and calculated a decrease in excess fatal cancer when this procedure was preferred over CT scans [8]. The availability and higher costs of MRIs may be the main reasons why the move to MRIs is slower in countries with fewer resources. Fortunately, in our department, MRIs are available, even on weekends. We are aware that this is not the case in all health systems and departments and would like to emphasize the relevance of availability for emergency MRI examinations in pediatric care.

**Figure 4:**
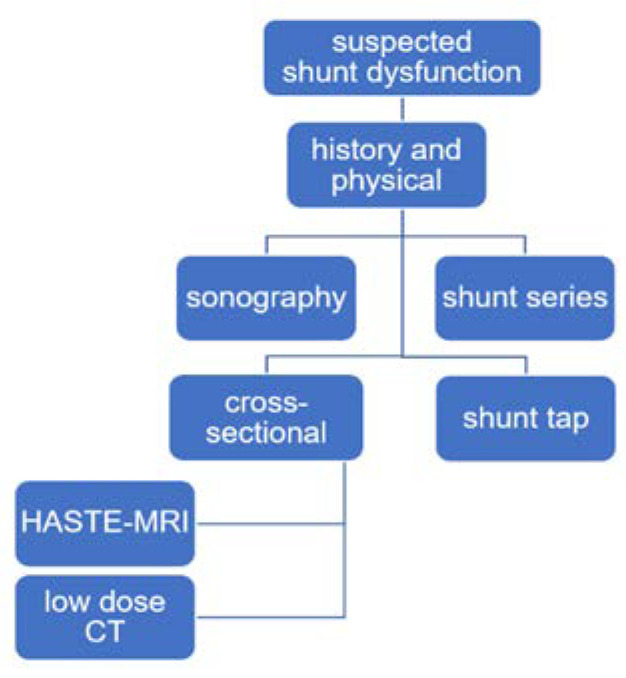
Clinical pathway for suspected shunt dysfunction as used in our department. Second line: For every patient, historical and physical examinations are performed. Third line: Sonography is performed for children if anatomically possible. Shunt series are routinely performed. Fourth line: As a cross-sectional imaging procedure, HASTE-MRI is the preferred modality. If not possible due to availability or patient characteristics, a low-dose CT scan is used. Shunt taps are routinely used to start CSF culture and verify shunt continuity.

When MRI is not available, other modalities might be useful in order to avoid CT scans. Ultrasound is known to be a risk-free diagnostic tool that can be used when the anterior fontanelle is still open. Mandiwanza et al. published a retrospective study in which they emphasize the use of ultrasound and its feasibility in diagnosing shunt malfunction [19]. Low-dose CT protocols reduce the radiation dose but also the image quality. Udayasankar et al. proved in their work that, despite a decrease in image quality, there is no decrease in diagnostic value and no necessity for repeat studies [20]. Another way to reduce CT use is the implementation of a clinical pathway that standardizes the use of diagnostic modalities in the emergency setting for shunt malfunction. Following such an implementation, Marchese et al. describe a reduction of over 50% for the average effective CT dose in an emergency department, without any increase in the 72 h revisit rate or CT use after readmission [21]. Figure 4 illustrates the simplified pathway for cases of suspected shunt dysfunction that is applied in our institution.

### Limitations

Our study has several limitations. Despite utilizing the largest health care institution in the area, the number of patients included was low. As to the nature of the study, since we included both the formerly used CODMAN shunt system and the proGAV shunt system introduced in 2017, patients characteristics such as mean shunt years per patient (11.37 vs. 2.28 years) and overall shunt years (295.8 vs. 34.3 years) were not equal. Therefore, we decided to limit direct the comparison between the two shunt systems to the first year of life. The exact tissue radiation doses per CT scan and X-ray differ for each scan and X-ray and were not available for this study. Therefore, an estimation of the doses was used, which could have led to over- or underestimation of the real radiation burden. We were not able to find reliable pediatric radiation doses for the standard diagnostic procedures, and we thus relied on adult doses. The actual dose might therefore have been underestimated.

## 5. Conclusions

Our results show that, by tackling radiation from different angles, such as substituting cCTs with MRIs and avoiding shunt series, additional radiation can be limited and even completely avoided after shunt insertion and the first year of life. We can therefore recommend the use of MRIs in routine follow-ups and suitable emergency situations. We also recommend using shunt systems with settings that can be verified without using lateral skull X-rays to further limit radiation exposure.

When evaluating the risk for radiation sequelae, we emphasize taking in consideration not just the diagnostics indicated by hydrocephalus but also those indicated by comorbidities, as these add to the overall burden. As the overall radiation burden in hydrocephalus patients is very specific to individuals, follow-ups and possible screenings for radiation sequelae should therefore be equally individualized. We emphasize the importance of larger trials covering a longer period in which MRI is used as a diagnostic modality in shunt treatment to evaluate safety and possible financial downsides.

## Figures and Tables

**Table 1:** This table shows the numbers of different diagnostic modalities per patient and per shunt year. It also provides an overview of the irradiation caused due to hydrocephalus and comorbidities per patient and per shunt year. cCT: cranial computertomography, MRI: magnetic resonance imaging.

	Rate per Patient (Range)	Rate per Shunt Year
cCT	1.9 (0–12)	0.23
Lateral skull X-ray	2.37 (0–13)	0.29
Shunt series	0.92 (0–4)	0.11
MRI head scan	6.63 (0–17)	0.82
	mSv per patient (range)	mSv per shunt year
Hydrocephalus-associated radiation	3.93 (0–24.38)	0.49
Comorbidity-associated radiation	4.3 (0–80.8)	1.85

**Table 2:** Comparison with previous studies. Values represent the numbers of imaging procedures per shunt year published by Antonucci et al., White et al. and Dobson et al. compared to our data. White et al. and Dobson et al. only investigated CT scans; therefore, no data were available for the other diagnostic modalities.

Study	Patients	Shunt Years	cCTs per Shunt Year	Shunt Series per Shunt Year	Skull X-rays per Shunt Year	Head MRIs per Shunt Year
Present study	41	330	0.23	0.12	0.37	0.68
Antonucci et al.	130	1300	0.9	0.3	0.1	0.1
White et al.	62	989	0.97			
Dobson et al.	152	778	0.65			

## Data Availability

Not applicable.
